# Structural atrophy and functional dysconnectivity patterns in the cerebellum relate to cerebral networks in svMCI

**DOI:** 10.3389/fnins.2022.1006231

**Published:** 2023-01-11

**Authors:** Alaka Acharya, Peng Ren, Liye Yi, Weiming Tian, Xia Liang

**Affiliations:** ^1^School of Life Science, Harbin Institute of Technology, Harbin, China; ^2^Department of Neurosurgery, The Second Affiliated Hospital of Harbin Medical University, Harbin, China; ^3^Laboratory for Space Environment and Physical Sciences, Harbin Institute of Technology, Harbin, China

**Keywords:** subcortical vascular cognitive impairment, basal ganglia, cerebellum, gray matter, voxel-based morphometry

## Abstract

Subcortical vascular mild cognitive impairment (svMCI) is associated with structural and functional changes in the cerebral cortex affecting major brain networks. While recent studies have shown that the intrinsic cerebral connectivity networks can be mapped onto the cerebellum, and the cortex and cerebellum are interconnected *via* the cortico-basal ganglia-cerebellar circuit, structural and functional disruptions in cerebellum in svMCI are rarely studied. In this study, we conducted voxel-based morphometry analysis to investigate gray matter atrophy pattern across cerebellar regions in 40 svMCI patients, and explored alterations in functional connectivity between the basal ganglia and cerebellum. The results showed that the amount of cerebellar atrophy within the default mode, salience, and frontoparietal networks correlated with their counterpart in the cerebral cortex. Moreover, key regions of the cerebellum, including the lobule VI, VIIb, VIII, and Crus I, which are reported to have a role in cognitive function, showed both anatomical atrophy and decreased functional connectivity with the striatum. These atrophy and connectivity patterns in the cerebellum also correlated with memory performances. These findings demonstrate that there are coupled changes in cerebral and cerebellar circuits, reflecting that degeneration patterns in svMCI are not limited to the cerebral cortex but similarly extend to the cerebellum as well, and suggest the cortico-basal ganglia-cerebellar circuit may play an important role in the pathology of svMCI.

## 1. Introduction

Subcortical vascular mild cognitive impairment (svMCI) is associated with structural and functional changes in the cerebral cortex affecting major brain networks. Despite traditional views suggesting that the cerebellum is engaged primarily in motor functions, recent studies suggest that the cerebellum contributes to higher-order brain function (Schmahmann and Sherman, 1998; Schmahmann, 2001). The cerebellum is structurally connected to distinct cerebral regions through polysynaptic fiber connections (Kelly and Strick, 2003; Buckner et al., 2011), and is also known to have functional associations with association cortex areas that are involved in cognitive processes (Habas et al., 2009; Krienen and Buckner, 2009; O'Reilly et al., 2010). Recent studies have shown that most cerebral intrinsic connectivity networks can be mapped onto the cerebellum (Habas et al., 2009; Buckner et al., 2011), which poses the question of whether the network degeneration observed in the cerebral cortex in svMCI can also be mapped onto the cerebellum.

The interconnections between the basal ganglia, cerebral cortex, and cerebellum are well established (Bostan et al., 2018; Milardi et al., 2019). Non-human studies along with neuroimaging studies in humans have accumulated evidence showing that the basal ganglia, the cerebellum, and the cerebral cortex are part of an interdependent integrated network (Caligiore et al., 2017; Bostan et al., 2018). This integrated network perspective of a co-dependent integrated network opens new avenues for the study of a basal ganglia-cortico-cerebellar network (Bostan and Strick, 2018; Bostan et al., 2018). Cerebellum regions are shown to be associated with reward-based learning similar to those observed in basal ganglia (Garrison et al., 2013). Several studies of human skill learning have shown that the interconnected basal ganglia-cortico-cerebellar network shows coordinated changes in activation throughout learning (Doyon and Benali, 2005; Lehéricy et al., 2005; Seidler et al., 2006). However, the roles of the basal ganglia-cortico-cerebellar network in neurodegenerative disorders affecting learning and memory have not been widely studied. In our previous study (Acharya et al., 2019), we revealed alterations in functional connectivity in svMCI between specific basal ganglia nucleus and widespread cortical areas, demonstrating an important role of a basal ganglia-cortex circuit in svMCI. However, it remains unclear whether and how the basal ganglia-cerebellar network alters in svMCI, and whether the changes in cerebellum couple with cortical dysfunctions.

Following these studies and also our previous work (Acharya et al., 2019) which focused on the basal ganglia-cortical connectivity in svMCI patients, we sought to study structural and functional connectivity changes in the cerebellum in svMCI patients by assessing gray matter volume changes across cerebellum, as well as functional connectivity of the basal ganglia with the cerebellum. Furthermore, we investigated whether the connectivity and atrophy pattern in the cerebellum correlates with cerebral connectivity and atrophy within the same network. Additionally, we also examined whether and how these structural and functional changes in the cerebellum are involved in learning and memory.

## 2. Materials and methods

### 2.1. Demographical information

Eighty right-handed participants, including forty svMCI patients and forty healthy controls, participated in this study and were matched on age, sex, and years of education. The patients were outpatients who were registered at the neurology departments of XuanWu Hospital, Capital Medical University, Beijing, China. This study was approved by the medical research ethics committee and institutional review board of Xuanwu Hospital, Capital Medical University, Beijing, China, and written informed consent was obtained from each participant.

The diagnosis of svMCI was performed by two experienced neurologists in consensus according to criteria (Erkinjuntti et al., 2000; Román et al., 2002; Petersen, 2004; Moorhouse and Rockwood, 2008) that included the following: (1) subjective cognitive complaints reported by the participant or his/her caregiver; (2) objective cognitive impairments, although not meeting the Diagnostic and Statistical Manual of Mental Disorders, fourth edition (DSM-IV) criteria for dementia; (3) normal or near-normal performance of general cognitive functioning and no or minimum impairments of daily life activities; (4) a Clinical Dementia Rating Scale (CDR) score = 0.5; (5) a Mini-Mental State Examination (MMSE) score ≥24 for middle school-educated, ≥20 for primary school-educated, and ≥17 for illiterate participants) (Zhang et al., 1990); and (6) subcortical vascular causes of the cognitive impairments according to a) moderate to severe white matter (WM) hyperintensity in at least one region with a Wahlund rating scale score ≥2 (Wahlund et al., 2001) and/or multiple lacunar infarcts in the periventricular and deep WM structures (Wahlund rating scale score ≥2; diameter,15 mm) on T2-weighted or FLAIR images, and b) evident neurological signs of hemiparesis, lower facial weakness, Babinski sign, dysarthria, sensory deficit, gait disorder, urgent urination or motor slowness that were assessed by general and neurological examination or reported by the participant or his/her caregiver. The exclusion criteria for svMCI included (Román et al., 2002; Zhou and Jia, 2009): (1) deficits in memory and other cognitive functions in the absence of focal lesions on brain imaging; (2) cognitive impairments as a result of other causes, such as a tumor, epilepsy, traumatic brain injury, multiple sclerosis, psychiatric disease, systemic disease (e.g., thyroid dysfunction, severe anemia, syphilis, and HIV), alcohol or drug abuse; (3) suffering of visual abnormalities, severe aphasia or palsy that made clinical assessments infeasible; (4) signs of large vessel diseases, such as cortical and/or cortico subcortical non-lacunar territorial infarcts and watershed infarcts or hemorrhages; and (5) diseases that led to white matter lesions, such as normal pressure hydrocephalus, multiple sclerosis, sarcoidosis or brain irradiation.

### 2.2. Data acquisition

All images were acquired using a 3.0 T Siemens scanner at XuanWu Hospital, Capital Medical University. Structural images consisted of sagittal magnetization-prepared rapid gradient echo (MP-RAGE) T1-weighted and T2-weighted sequences. The T1-weighted sequence had the following parameters: repetition time [TR] = 1,900 ms; echo time [TE] = 2.2 ms; inversion time [TI] = 900 ms; flip angle [FA] = 9°; number of slices = 176; slice thickness = 1.0 mm; data matrix = 256 × 256; field of view [FOV] = 256 × 256 mm^2.^ The T2-weighted images had the following parameters: TR = 4040 ms; TE = 84 ms; FA = 160°; number of slices = 20; slice thickness = 5.0 mm; gap = 6.5mm; data matrix = 320 × 186; FOV = 240 × 140 mm^2^. Resting-state functional images were acquired using an echo-planar imaging sequence, and acquisition parameters are: TR = 2,000 ms; TE = 40 ms; FA = 90°; number of slices = 28; slice thickness = 4 mm; gap = 1 mm; data matrix = 64 × 64; FOV = 256 × 256 mm^2^. The subjects were instructed to lie quietly in the scanner with their eyes closed and to remain stable as much as possible during the data acquisition. The functional scan lasted for 478 s (239 volumes) in total. During the scan, foam pads and headphones were used to reduce head motion and scanner noise as much as possible.

### 2.3. Voxel-based morphometry analysis

T1-weighted images were segmented into gray and white matter using the VBM toolbox in SPM12. The output images were visually inspected for each participant to ensure accurate segmentation. The gray matter images were further normalized into Montreal Neurological Institute (MNI) space (affine + non-linear), modulated (non-linear only), and smoothed with a 8 mm full width at half maximum Gaussian kernel. For the normalization, the Dartel toolbox in SPM12 was used.

The cerebellum was processed using the spatially unbiased infratentorial template (SUIT) toolbox specifically developed for the cerebellum (Diedrichsen, 2006). Compared with the standard whole-brain atlases, the high-resolution atlas used by SUIT preserves the anatomical detail of the cerebellum and provides more accurate spatial registration (Diedrichsen, 2006). The cerebellum was first isolated using a stepwise Bayesian algorithm that estimates the likelihood of each voxel belonging to the cerebellum, normalized to the MNI space with the high-resolution probability cerebellum template in SUIT (affine + non-linear), modulated, and smoothed with a 2 mm full-width at half-maximum Gaussian kernel. In both cases, non-linear modulation was selected as it y allows comparison of the absolute amount of tissue corrected for individual brain sizes.

### 2.4. Seed-based functional connectivity analysis

Functional MRI images were pre-processed using the Analysis of functional neuroimaging software (Cox, 1996). The pre-processing steps consisted of slice timing correction, motion correction, spatial smoothing (FWMH = 6 mm), band-pass temporal filtering (0.01–0.1 Hz), and spatial normalization to standard Talairach space and removal of the head motion profiles, the white matter, and CSF signal. To moderate the effects of head motion on estimates of resting-state functional connectivity (rsFC) (Power et al., 2012; Satterthwaite et al., 2012; van Dijk et al., 2012), we first calculated the average root mean square (RMS) of head motion and found no significant between-group difference (F = 1.15, *P* = 0.32). The average RMS of head movement was considerably below the cutoff of 1 mm (average RMS = 0.13 for NC and 0.14 for SVMCI). Secondly, we censored volumes within each subject's fMRI time series that were associated with sudden head movements. For each subject, fMRI volume was censored if its frame-wise displacement (FD) > 0.35. In average, 12 volumes were censored across all subjects.

We selected the caudate nucleus, the key basal ganglia structure, which showed the maximum structural and functional impairment in our previous report (Acharya et al., 2019), as the Region of Interest (ROI) to calculate the functional connectivity of both cortex and cerebellum. The caudate was defined according to the Human Brainnetome Atlas (Fan et al., 2016), which was also used in our previous work. We computed the rsFC of the caudate regions as follows. Within the seed region, we calculated the Pearson correlations with Fisher's z-transformation between the averaged time courses of the seed region and all other brain voxels. Within-group one-sample *t*-test was performed for the seed region and a rsFC map for each group was created by applying a threshold of *p* < 0.0001 with a cluster size of 86 voxels (*P*_corrected_ < 0.001 based on Monte Carlo simulations).

### 2.5. Statistical analysis

Group differences in demographics were evaluated using two-sample *t*-tests for continuous variables, and a Chi-square test for categorical variables.

To examine the group differences in the gray matter intensity between svMCI and healthy controls, the output of VBM for cortex and cerebellum was subjected to two-sample *t*-tests, respectively. A voxel-wise threshold of *P* < 0.01 combined with a cluster threshold of 86 voxels (*P*_*corrected*_ < 0.05) as derived from Monte Carlo Simulation was used to obtain the group difference map. A similar *t*-test analysis was done to explore caudate rsFC differences between SVMCI and NC groups across the cortex and the cerebellum, respectively. To improve the sensitivity of detection while still controlling for the false positive rate, a voxel-wise threshold of *P* < 0.01 combined with a cluster threshold of 60 voxels (*P*_*corrected*_ < 0.05) as derived from Monte Carlo Simulation was used to obtain the group difference map, with the restriction that significant clusters must belong to the “OR” rsFC map of the seed region in at least one group. The resulting group difference maps were compared with the Buckner and Yeo atlas of the cerebellum and the cerebrum respectively (Buckner et al., 2011; Yeo et al., 2011) to find out the atrophy and functional dysconnectivity patterns across different brain networks. To investigate the relationship between the cortex and cerebellum, we conducted correlation analyses Gray Matter intensity or caudate functional connectivity across the brain networks between the cortex and cerebellum. Age and gender were included as covariates in the group level comparisons.

## 3. Results

### 3.1. Demography and behavior

Table 1 shows the demographic data of the healthy controls and the patients with svMCI. Both groups were matched in terms of age [F_(2, 78)_ = 0.025, *P* = 0.97], gender (χ^2^ = 0.625, *P* = 0.73) and years of education [F_(2, 78)_ = 1.14, *P* = 0.32]. Moreover, there were significant group differences in MMSE [F_(2, 78)_ = 19.7, *P* < 0.001], AVLT-immediate [F_(2, 78)_ = 32.6, *P* < 0.001], AVLT-delayed recall [F_(2, 78)_ = 48.1, *P* < 0.001] and AVLT-recognition scores [F_(2, 78)_ = 22.4, *P* < 0.001].

**Table 1:** Demographic characteristics of the participants.

	**NC (*n* = 40)**	**svMCI (*n* = 40)**	***P*-Value**
Gender (M/F)	15/25	18/22	0.73
Age	64.67 ± 5.8	64.4 ± 9.3	0.97
Education years	10.6 ± 5.25	8.975 ± 4.75	0.32
MMSE	28.2 ± 2.1	25.575 ± 3.6	< 0.001^*^
AVLT-immediate recall	9.075 ± 1.6	7 ± 2.6	< 0.001^*^
AVLT-delayed recall	9.925 ± 2.6	7.025 ± 3.07	< 0.001^*^
AVLT-recognition	11.975 ± 2.7	10.5 ± 2.5	< 0.001^*^

* A significant difference across the three groups. n, number of subjects; NC, normal control; svMCI, subcortical vascular mild cognitive impairment group; MMSE, Mini-Mental Status Examination; AVLT, Auditory Verbal Learning Test. Means ± standard deviation.

### 3.2. Gray matter volume distribution in cortical network mirrors cerebellar network

**Figure 1:**
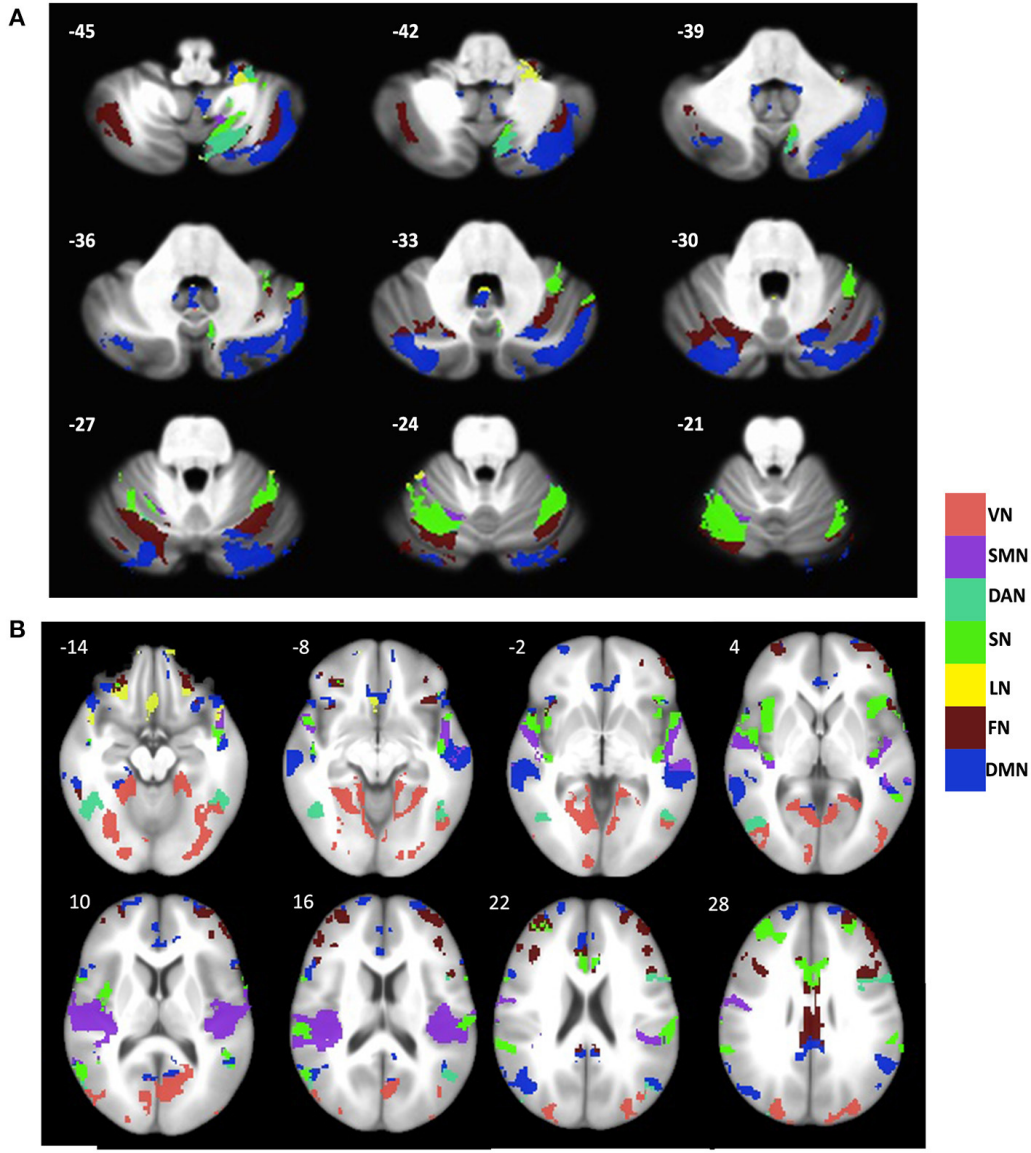
Distribution of gray matter atrophy pattern across major brain networks in the **(A)** cerebellum and **(B)** cerebral cortex in svMCI. The brain network parcellation was adopted from Buckner atlas for cerebellum (Buckner et al., 2011) and Yeo atlas for cerebral cortex (Yeo et al., 2011). VN, Visual network; SMN, Somatomotor Network; DAN, Dorsal Attention Network; SN, Salience Network; LN, Limbic Network; FN, Frontal Network; DMN, Default Mode Network.

In the cerebrum, the gray matter atrophy in svMCI was observed in regions such as bilateral angular gyrus, posterior cingulate cortex/precuneus, anterior cingulate cortex, and bilateral insula, which are key regions of the default network and salience network. Additionally, brain regions including the precentral and postcentral gyrus, bilateral middle frontal gyrus, and temporal gyrus, showed significant between-group differences in gray matter intensity. These regions are distributed predominately in the somatomotor, frontoparietal, and dorsal Attention networks as classified according to the Buckner atlas (Figure 1A).

In Cerebellum, regions including the inferior semilunar lobule (lobule VIIIa Crus I), lobule VIIa Crus I, Crus I, VI, vermis XI/X, lobule IX, lobule X showed major group differences in gray matter intensity. These regions encompass all seven networks. As seen in the cerebral cortex, the regions within the default network, salience network, and frontoparietal network showed the most prominent changes in gray matter intensity (Figure 1B).

**Figure 2:**
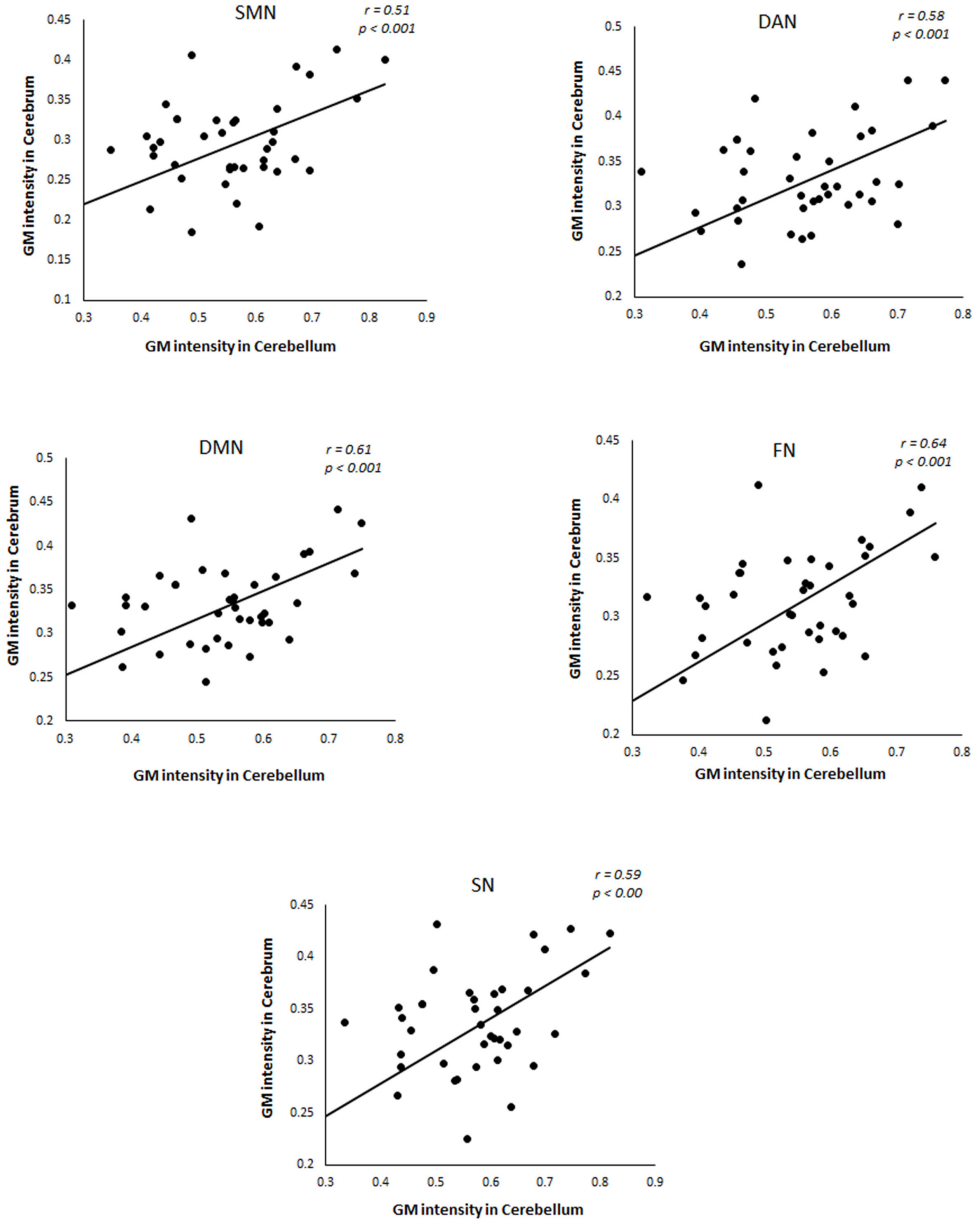
Relationship between GM intensity in cerebral networks and its cerebellar counterparts in svMCI patients. SMN, Somatomotor Network; DAN, Dorsal Attention Network; DMN, Default Network; FN, Frontal Network; SN, Salience Network.

If the variations in large-scale brain networks observed in svMCI extend to the cerebellum, the atrophy pattern in the cerebral network should mirror their cerebellar counterpart. To test this hypothesis, we compared the gray matter difference maps between previously established cerebral and cerebellar networks (Buckner et al., 2011; Yeo et al., 2011). Our results showed that in svMCI, all the corresponding brain networks showed coupled changes in the gray matter atrophy pattern with the exceptions of the visual and limbic networks (Figure 2).

### 3.3. Similar connectivity pattern of caudate within cerebral and cerebellar networks

**Figure 3:**
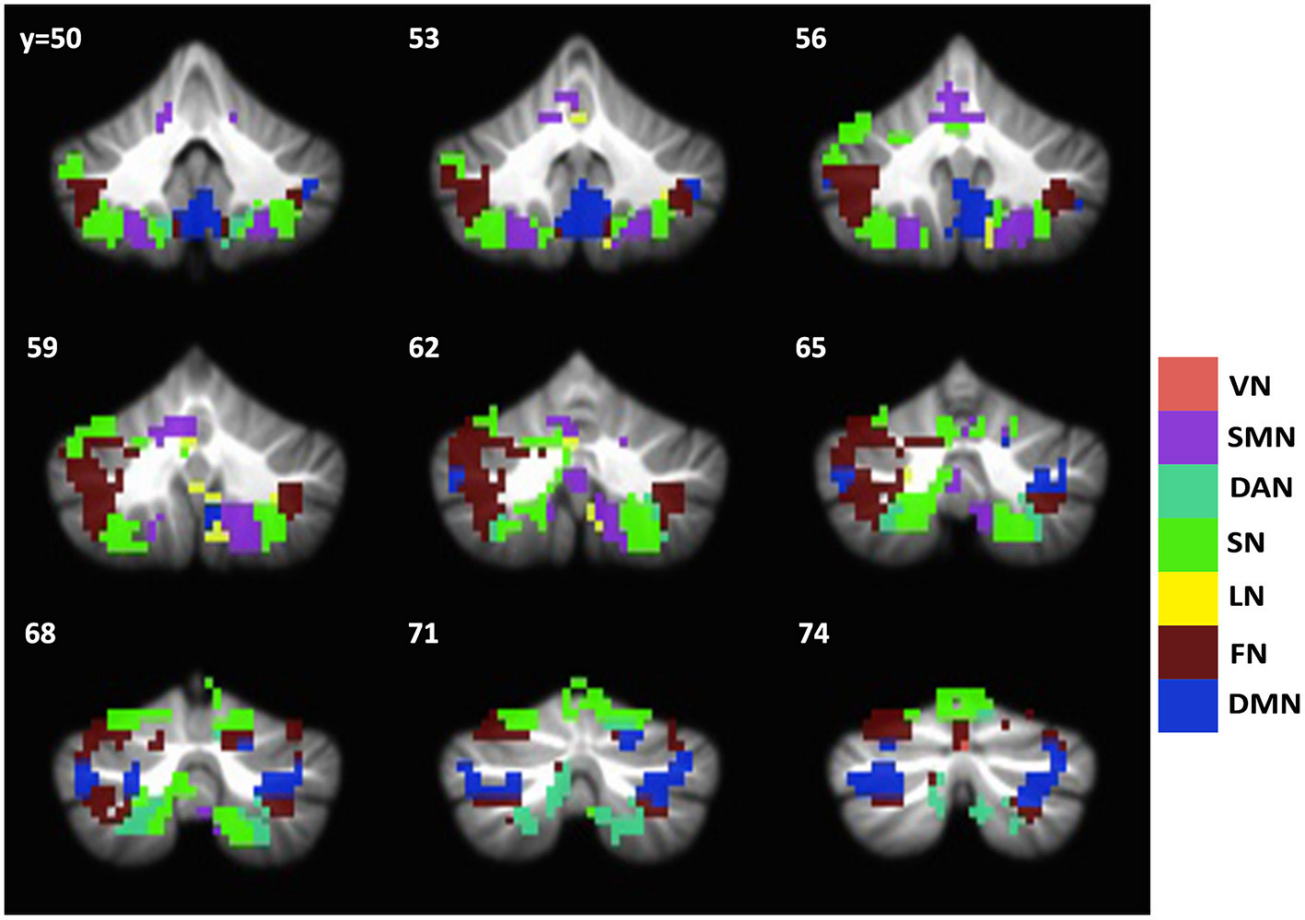
Group difference map of caudate functional connectivity between svMCI and NC groups in the cerebellum. Brain regions showing significant group difference are distributed across all seven networks. The group difference map of cerebellum is overlayed with Buckner atlas for cerebellum. VN, Visual network; SMN, Somatomotor Network; DAN, Dorsal Attention Network; SN, Salience Network; LN, Limbic Network; FN, Frontal Network; DMN, Default Mode Network.

**Figure 4:**
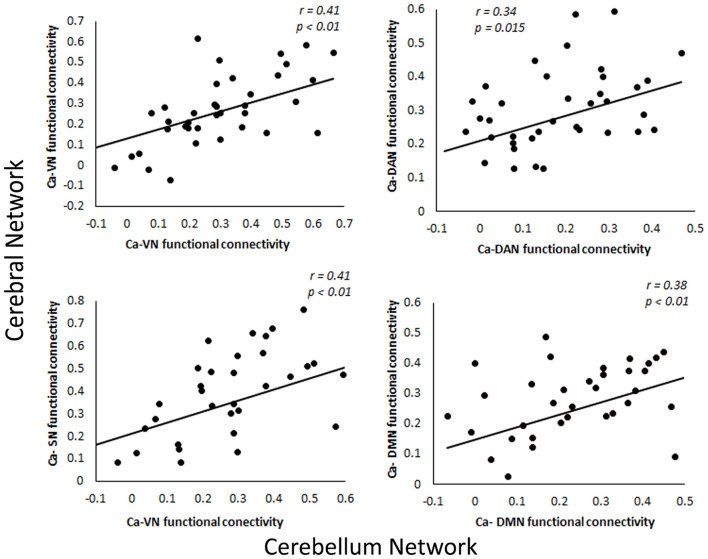
Relationship between functional connectivity of caudate in cerebral and cerebellar networks. VN, Ventral Network; DAN, Dorsal Attention Network; SN, Somatomotor Network; DMN, Default Mode Network.

Since the caudate nucleus is the most impaired basal ganglia structure in terms of cortical functional connectivity in our previous studies (Acharya et al., 2019), we selected the caudate nucleus as the seed region to evaluate functional connectivity changes with the cerebellum regions in svMCI. We found differences in the functional connectivity of the caudate between the two groups in regions including the bilateral Crus I, VI, Crus II, VIIIa, VIIb, X, and IV. These regions were predominately distributed in the frontoparietal network, salience network, and default network (Figure 3). To test if the functional connectivity of the caudate across the various network in the cerebral cortex and the cerebellum correlate with each other, especially among similar networks, correlation analysis was performed. Four sets of significant relationships were observed. Functional connectivity between the caudate with the visual (r = 0.41, *p* < 0.01), salience (r =0.41, *p* < 0.01), dorsal Attention (r = 0.34, *p* = 0.015) and default network (r = 0.38, *p* < 0.01) in the cerebellum correlated significantly with their cerebral counterpart (Figure 4).

### 3.4. Structural and functional changes in the cerebellum correlate with cognitive impairments in svMCI

**Figure 5:**
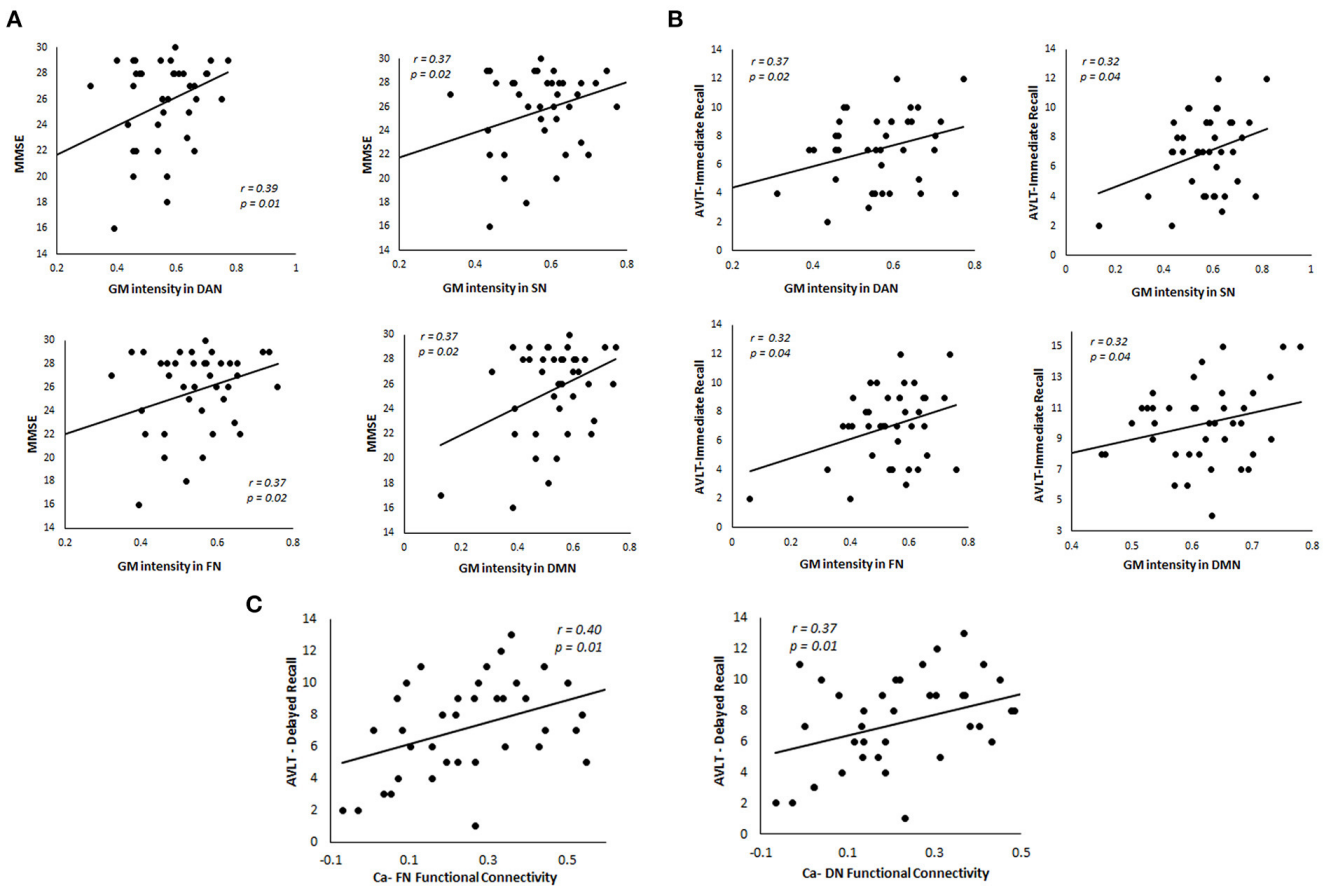
Relationship between GM intensity and caudate functional connectivity in the cerebellum in svMCI patients. **(A)** Correlation between MMSE scores and GM intensity in four major cerebellar networks. **(B)** Relationship between AVLT-Immediate Recall performances and GM intensity in four major cerebellar networks. **(C)** Relationship between functional connectivity of caudate with cerebellar networks and AVLT-Delayed Recall performances in svMCI patients. FN, Ventral Network; DAN, Dorsal Attention Network; SN, Somatomotor Network; DMN, Default Mode Network; Ca, Caudate.

We performed regression analysis to understand the relationship between gray matter intensity and learning and memory in svMCI. In the cerebellum, the intensity values in the dorsal Attention network, salience network, frontoparietal network, and default mode network correlated with both MMSE (Figure 5A) and AVLT immediate recall (Figure 5B). We also investigated the relationship between caudate-cerebellar functional connectivity and memory performances in svMCI. The functional connectivity of caudate with frontoparietal network (r = 0.40, *p* = 0.01) and default network (r = 0.37, *p* = 0.01) showed significant correlations with AVLT delayed recall performances (Figure 5C). Two subjects were excluded as they were considered outliers which did not impact the correlation between the variables.

## 4. Discussion

Our findings showed that neurodegenerative symptoms in subcortical vascular mild cognitive impairment are associated with coupled changes in structural and functional integrity in the cerebellum and the cerebral cortex. We found distributed gray matter atrophy in both cerebrum and cerebellum in svMCI, which showed a strong correlation in gray matter intensities across brain networks between cerebrum and cerebellum. Similarly, between-group changes in functional connectivity with the caudate nucleus of the basal ganglia were similar across cerebellar networks and their cerebral counterparts. Also, in svMCI, the GM intensities in the dorsal attention, salience, default mode, and frontoparietal networks in the cerebellum correlated with immediate verbal recall performance and MMSE. Moreover, the functional connectivity of caudate with frontoparietal and default networks showed significant correlations with delayed recall performances.

The contribution of basal ganglia in neurodegenerative symptoms in the cerebral cortex has been widely established and its contribution and involvement in svMCI have been studied in our previous study (Acharya et al., 2019). Increasing new evidence has shown that a direct cerebellar-basal ganglia connection is involved in cognition, emotion, and several other complex tasks (Schmahmann and Caplan, 2006; Milardi et al., 2019). Basal ganglia and cerebellum connectivity is found to be enhanced at the beginning of the learning process (Sami et al., 2014). A pathway projecting the cerebellum to and from basal ganglia has been identified in humans and non-human primates using anterograde and retrograde studies (Bostan et al., 2010). Since the caudate is the associative area of the striatum, it's been known to have connections to the areas responsible for higher-order executive functions in the cerebellum and the cerebral cortex (Habas et al., 2009; Habas, 2021).

Our study is one of the very few so far to systematically examine cerebellar atrophy in relation to intrinsic brain networks in svMCI. Among the cerebellar subregions targeted by svMCI, areas such as lobule VIIb, VIII, bilateral Crus I, and VI, showed both structural loss and decreased functional connectivity to the caudate. Studies have shown that these regions, which belong to somatomotor, salience, and default mode networks and project to the prefrontal cortex (cerebello-thalamo-cortical loop), contribute to higher-order cognition in the cerebellum and are activated during cognitive and working memory-related tasks (Kelly and Strick, 2003, Stoodley and Schmahmann, 2009; Marvel and Desmond, 2010; Stoodley, 2012; Stoodley et al., 2012; Buckner, 2013). Lesions in lobules VI and VII have been found to be responsible for impairments in cognition by interrupting the cerebrocerebellar cognitive loops (Stoodley and Schmahmann, 2010). In a study where attention training was given to mild cognitive impairment patients who also had subcortical vascular changes, these cerebellar areas showed increased activity after the treatments were given (Pantoni et al., 2017). One study in patients with svMCI showed that the decreased homogeneity of Crus I attributed to the decline in cognitive ability (Diciotti et al., 2017). These evidences are in line with our current findings that both structure and functions of these cerebellar regions are affected/involved in svMCI.

The findings from this study present evidence that the basal ganglia which are known to have interconnections with functionally related areas of the cerebral cortex and cerebellum are involved in the pathology of svMCI. This interconnection between the caudate, which is the associative territory of the basal ganglia, dorsolateral prefrontal cortex, and Crus I and Crus II of the cerebellum is known to be associated with higher order executive function and working memory (Habas et al., 2009). Cognitive dysfunction in several neurodegenerative disorders is associated with abnormal activity within this network (Caulfield et al., 2016; Pereira et al., 2017; Zheng et al., 2017). The decreased functional connectivity of the caudate nucleus to these aforementioned regions observed in our study is consistent with these findings, which further solidifies that similar abnormality in the larger interconnected network is observed in svMCI as well.

There are multiple evidences of cerebrocerebellar circuits that are involved in higher cognitive functions such as attention, executive control, working memory, and learning (Ramnani, 2006; Strick et al., 2009). A recent study has shown that a cognitive network consisting of the dorsolateral prefrontal cortex, dorsomedial striatum, and lateral posterior cerebellum is involved in model-based learning (Fermin et al., 2016). The correlations between cognitive performances and the functional connectivity of the caudate with the cerebral cortex and cerebellum seen in this study provide further evidence for the existence of a cortico-basal ganglia-cerebellar network responsible for learning and memory, which are typically affected in svMCI.

Our results indicate that cerebellum has an important contribution to memory and cognition and hence point toward the importance of focused cerebellar studies to better understand the pathophysiology of neurodegeneration. These raise interesting and intriguing questions regarding the contribution of the cerebellum to cognitive symptoms and indicate that more studies dedicated to the cerebellum and its interconnections to the cortical and subcortical areas are required in order to better understand the pathophysiology of neurodegenerative disorders.

Despite promising results, there are several limitations to our findings. In this study, the functional connectivity study of the cerebellum was focused on a subsection of the basal ganglia nuclei, the caudate region. Expanding the region of interest to the broader structures within the basal ganglia can give a clearer picture regarding the BG-Cerebellar circuit in neurodegeneration. Furthermore, whole-brain connectivity analysis using cerebellar and cerebral regions as regions of interest simultaneously could provide better insights regarding the cerebellar involvement in cognition and thereby provide a better understanding of the cortico-basal ganglia-cerebellar circuit.

## Data availability statement

The raw data supporting the conclusions of this article will be made available by the authors, without undue reservation.

## Ethics statement

The studies involving human participants were reviewed and approved by Medical Research Ethics Committee and institutional review board of Xuanwu Hospital, Capital Medical University, Beijing, China. The patients/participants provided their written informed consent to participate in this study. Written informed consent was obtained from the individual(s) for the publication of any potentially identifiable images or data included in this article.

## Author contributions

AA, XL, and LY designed research. AA performed research. AA and PR analyzed data. AA, XL, WT, and LY wrote the paper. All authors contributed to the article and approved the submitted version.
